# Design, Synthesis and Gene Modulation Insights into Pigments Derived from Tryptophan-Betaxanthin, Which Act against Tumor Development in *Caenorhabditis elegans*

**DOI:** 10.3390/ijms25010063

**Published:** 2023-12-20

**Authors:** Paula Henarejos-Escudero, Fernando F. Méndez-García, Samanta Hernández-García, Pedro Martínez-Rodríguez, Fernando Gandía-Herrero

**Affiliations:** Department of Biochemistry and Molecular Biology A, Faculty of Biology, Regional Campus of International Excellence, Campus Mare Nostrum, University of Murcia, 30100 Murcia, Spain; paula.henarejos@um.es (P.H.-E.); fernandofederico.mendezg@um.es (F.F.M.-G.); samanta.hernandez@um.es (S.H.-G.); pedro.martinezr@um.es (P.M.-R.)

**Keywords:** betaxanthins, cancer, *Caenorhabditis elegans*, phytochemicals, preclinical model, tryptophan

## Abstract

The use of betalains, which are nitrogenous plant pigments, by the food industry is widespread and reflects their safety after intake. The recent research showed outstanding results for L-tryptophan-betaxanthin, a phytochemical present in traditional Chinese medicine, as an antitumoral agent when the activity was evaluated in the animal model *Caenorhabditis elegans*. Thus, L-tryptophan-betaxanthin is now presented as a lead compound, from which eleven novel structurally related betaxanthins have been designed, biotechnologically produced, purified, and characterized. The antitumoral effect of the derived compounds was evaluated on the JK1466 tumoral strain of *C. elegans*. All the tested molecules significantly reduced the tumoral gonad sizes in a range between 31.4% and 43.0%. Among the novel compounds synthesized, tryptophan methyl ester-betaxanthin and tryptophan benzyl ester-betaxanthin, which are the first betalains to contain an ester group in their structures, caused tumor size reductions of 43.0% and 42.6%, respectively, after administration to the model animal. Since these were the two most effective molecules, their mechanism of action was investigated by microarray analysis. Differential gene expression analysis showed that tryptophan methyl ester-betaxanthin and tryptophan benzyl ester-betaxanthin were able to down-regulate the key genes of the mTOR pathway, such as *daf-15* and *rict-1*.

## 1. Introduction

Despite of the many advances in medicine, cancer is still one of the major causes of mortality worldwide, with more than 19 million of cases diagnosed in 2020 [1]. This complex disease implies the uncontrolled growth of cells caused by the accumulation of defective genes which control the cellular cycle, among many other critical pathways, alongside with environmental factors [2,3,4]. Thus, novel treatments with enhanced or complementary effectivity and with fewer side effects are urgently needed because no definite new and safe remedies have been found in more than fifty years of molecular cancer research. Although some human cancers can be cured with mixed treatments and, in other cases, can prolong the patient’s life expectancy, they also present very detrimental side effects.

On the search for novel drugs for cancer treatment, betalains have shown promising results in recent years [5,6]. These are a family of plant pigments with a strong health-promoting potential [7], and among their numerous reported pharmacological effects, protection against oxidative [8,9,10,11] and inflammatory [12,13,14,15] processes is included. L-Tryptophan-betaxanthin is a betalain found, among other sources, in traditional Chinese medicine (TCM) plants [16,17,18,19,20,21]. It has been studied by different computational methods, showing interesting interactions with receptors and proteins. It exhibited a good binding affinity with the Silent information regulator 1 (Sirt1) protein. This protein is a member of the sirtuin family, which is important for regulating metabolic signaling pathways, and it is involved in the diseases associated with aging [22]. Tryptophan-betaxanthin has also been proposed as an inhibitor of the fat mass and obesity-associated protein (FTO) through competitive inhibition. The FTO protein is linked to numerous diseases, such as cardiovascular diseases, diabetes, and cancer [23]. In other in silico studies, tryptophan-betaxanthin has been identified as a potential agonist, targeting the peroxisome proliferator-activated receptor (PPAR) protein for treating metabolic syndromes, which are a collection of disorders characterized by obesity and multiple clinical disorders. Finally, this molecule was described by our group as a potent antitumoral drug after studies performed in vivo in the animal model *Caenorhabditis elegans* [24]. In that study, in addition to tryptophan-betaxanthin, the other individual betalains, such as betanin, phenylethylamine-betaxanthin, indicaxanthin, phenylalanine-betaxanthin, and dopaxanthin, were tested. Of all of them, the most effective molecule against tumor growth in nematodes was tryptophan-betaxanthin, which was chosen as the lead compound for the design and development of the new molecules showed in this work. Overall, the promising background of the betalain L-tryptophan-betaxanthin encourages the study of this betaxanthin as a lead molecule in cancer treatment [24].

The small nematode *Caenorhabditis elegans* has been proposed as an emerging model for cancer research. Although these models could not replace mammalian testing, they provide useful information on the underlying mechanisms of action of the tested compounds [25,26]. There are several *C. elegans* mutant strains associated with different human cancer types [27]. Among them, the JK1466 strain generates tumors in the germline which resembles teratomas in humans [28]. These tumors are usually located in women’s ovaries and in men’s testes. Teratomas could be lethal if not treated properly.

About some of the pathways involved in cancer, such as cell differentiation, proliferation, and survival. The mTOR (mechanistic target of rapamycin) signaling pathway plays a key role in it. Some of the genes of mTOR signaling pathway are *rict-1* and *daf-15*, the *C. elegans* orthologs to the human RICTOR (rapamycin-insensitive companion of mammalian target of rapamycin) and RAPTOR (regulatory-associated protein of mTOR), respectively. In cancerous cells, this pathway is frequently overactivated. In fact, the down-regulation of RICTOR and/or RAPTOR was shown to be a promising approach for cancer therapy [29]. Hou and co-authors reported that the knock-down of RICTOR enhanced the antitumoral effects of LY294002 (a phosphatidylinositol 3-kinase, PI3K, inhibitor) in esophageal squamous cell carcinoma [30]. In the same line, Zhu and co-authors showed that cardamomin reduced the proliferation of SKOV3 cells by suppressing the expression of RAPTOR [31].

Another mechanism of action for the progression of cancer is to avoid apoptotic cell death. The recent studies show that the down-regulation of caspase3 may be a survival mechanism for breast cancer [32] and prostate cancer cells [33]. Some genes encoding the caspases, *ced-3* and *csp-1*, are orthologs of the human caspases CASP3, CASP6, and CASP7 that promote apoptotic cell death. 

This paper presents the production, purification, and characterization of novel molecules derived from the phytochemical tryptophan-betaxanthin found in traditional Chinese medicine plants. Using L-tryptophan-betaxanthin as a lead compound, multiple novel compounds were rationally designed. The molecules were tested for antitumoral potential in vivo in the *C. elegans* cancer model JK1466, and the underlying mechanisms of action for the antitumoral effects were studied by differential gene expression analysis.

## 2. Results

### 2.1. Production and Purification of Tryptophan-Derived Betaxanthins

A biotechnological strategy was employed to produce the tryptophan-betaxanthin-derived pigments. The expression of the dioxygenase enzyme from *G. diazotrophicus* in the transformed *E. coli* was used to obtain betalamic acid from L-DOPA (7.6 mM) and 6-decarboxy-betalamic acid from dopamine (7.6 mM) [34]. Both the compounds were used as structural units to condense the molecules structurally related to the amino acid tryptophan for 72 h. The optimal concentration of these compounds added to the reaction medium was established at 10 mM in the preliminary experiments to improve the yield. Thus, using this approach, 11 different betalains were obtained, both pure L or D isomers and racemic mixtures. Each betaxanthin was derived from a different precursor molecule structurally related to tryptophan: L-tryptophan, D-tryptophan, tryptamine, 5-hydroxy-L-tryptophan, 5-fluoro-DL-tryptophan, 5-bromo-DL-tryptophan, L-tryptophan benzyl ester, L-tryptophan methyl ester, and serotonin.

**Figure 1:**
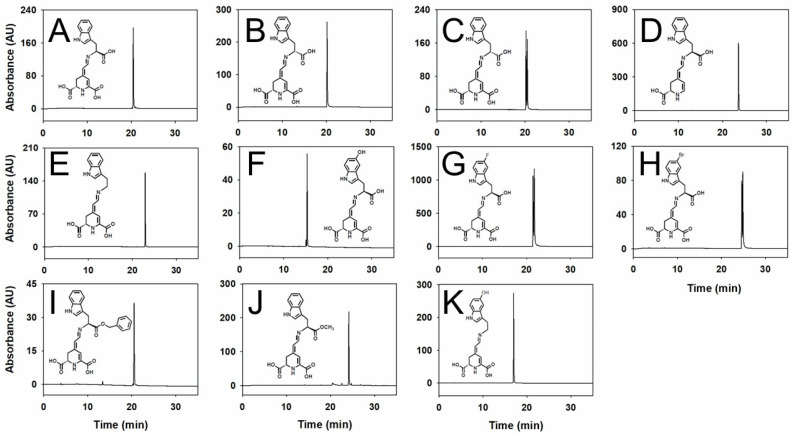
Structures and chromatograms of pure tryptophan-derived betaxanthins. HPLC chromatograms are shown at 480 nm of each individual betalain or racemic mixture. (**A**) L-tryptophan-betaxanthin, (**B**) D-tryptophan-betaxanthin, (**C**) DL-tryptophan-betaxanthin, (**D**) L-tryptophan-6-decarboxy-betaxanthin, (**E**) tryptamine-betaxanthin, (**F**) 5-hydroxy-L-tryptophan-betaxanthin, (**G**) 5-fluoro-DL-tryptophan-betaxanthin, (**H**) 5-bromo-DL-tryptophan-betaxanthin, (**I**) L-tryptophan-benzyl ester-betaxanthin, (**J**) L-tryptophan methyl ester-betaxanthin, and (**K**) serotonin-betaxanthin. The injection volume was 50 μL.

After, ion exchange chromatography and C-18 reversed-phase solid-phase extraction were applied to each betalain obtained by biotransformation, and HPLC analyses were performed to confirm the pigments purity (Figure 1). Panels C, G, and H show the analysis of the molecules that appeared clearly as two peaks corresponding to the diastereoisomeric forms of the pigments. These two forms are derived from the racemic mixtures of the corresponding amino acids. Zoomed-in images of these peaks are shown in Figure S1. Ten different yellow betaxanthins and one 6-decarboxy-betaxanthin with maximum absorbance values around 480 nm were obtained and analyzed by HPLC. All eleven compounds were analyzed by HPLC-ESI-TOF-MS to unambiguously identify the molecular mass and support the structural identification of the novel betaxanthin analog. The results confirmed the nature of the pigments proposed, as summarized in Table 1.

The HPLC retention times (Rt) of the betaxanthins obtained were 15.31 min for 5-hydroxy-L-tryptophan-betaxanthin, 16.97 min for serotonin-betaxanthin, 20.17 min for D-tryptophan-betaxanthin, 20.47 min for L-tryptophan-betaxanthin, 20.53 min for L-tryptophan-benzyl ester-betaxanthin, 21.53 and 21.78 min for 5-fluoro-DL-tryptophan-betaxanthin, 22.95 min for tryptamine-betaxanthin, 23.63 min for L-tryptophan-6-decarboxy-betaxanthin, 24.16 for L-tryptophan methyl ester-betaxanthin, and 24.63 and 24.68 min for 5-bromo-DL-tryptophan-betaxanthin (Figure 1 and Table 1).

### 2.2. Color of Tryptophan-Derived Betaxanthins

In general, betalains present a high absorbance level, and tryptophan-betaxanthin shows an intense yellow color. Thus, the absorbance of the novel betaxanthins rationally designed was studied and recorded for the first time (Table 2 and Figure S2). The maximum wavelengths obtained range from 475 to 479 nm. These pigments lack an indoline-like cycle in resonance with the betalamic acid or 6-decarboxy-betalamic acid moieties, and thus, they are yellow in color and can be considered betaxanthins derived from L-tryptophan amino acid. 

In addition, the molar absorption coefficients were calculated for all the pigments using an end-point degradation method. The values were high for all the molecules and ranged from 36,000 to 55,000 M^−1^·cm^−1^, as shown in Table 2. 

### 2.3. Effect of Tryptophan-Derived Betaxanthins on Tumor Growth in C. elegans

The JK1466 strain of *C. elegans* has the tumor suppressor gene *gld-1* knocked-down. This alteration causes the proliferation of cells in the gonad that ultimately produces a tumor which is lethal for the nematodes [35].

**Figure 2:**
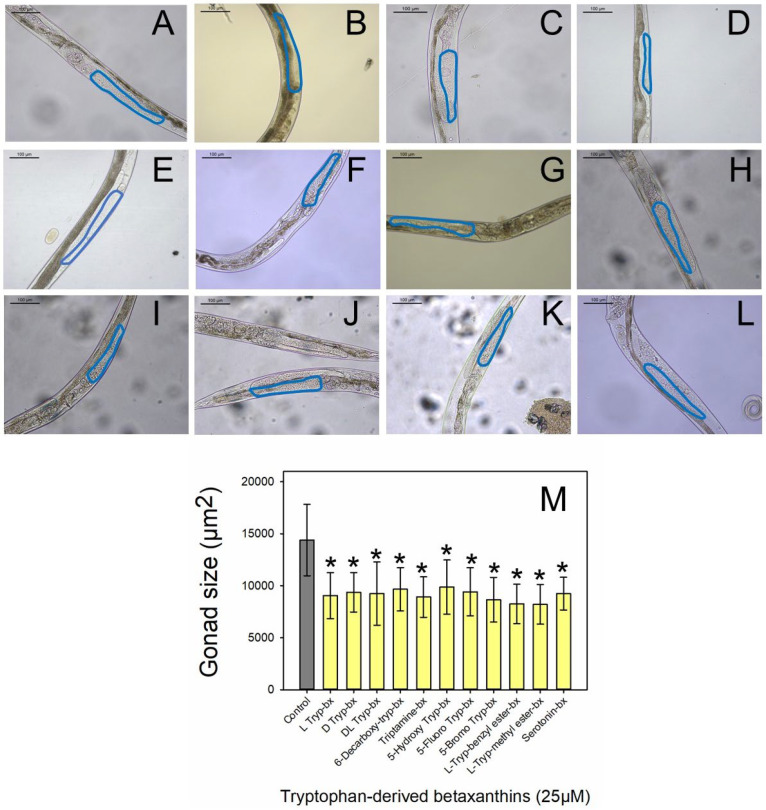
Sizes of tumoral gonads in *C. elegans*. Representative images of *C. elegans* JK1466 strain (**A**) without treatment (control) and with different treatments: (**B**) L-tryptophan-betaxanthin, (**C**) D-tryptophan-betaxanthin, (**D**) DL-Tryptophan-betaxanthin, (**E**) L-tryptophan-6-decarboxy-betaxanthin, (**F**) tryptamine-betaxanthin, (**G**) 5-hydroxy-L-tryptophan-betaxanthin, (**H**) 5-fluoro-DL-tryptophan-betaxanthin, (**I**) 5-bromo-DL-tryptophan-betaxanthin, (**J**) L-tryptophan-benzyl ester-betaxanthin, (**K**) L-tryptophan methyl ester-betaxanthin, and (**L**) serotonin-betaxanthin. Scale bar: 50 µm. (**M**) Gonad size histograms (mean values ± SD) of *gld-1* mutants control (n = 243) and treated with the different tryptophan-derived betaxanthins (n ≥ 56). bx: betaxanthin. * indicates a statistically significant difference in values (*p* ≤ 0.05) with respect to the data obtained for controls. Scale bar: 100 µm.

To examine the effect of the novel compounds synthesized from L-tryptophan-betaxanthin as a lead compound in cancer prevention or treatment, the gonad sizes of the nematodes were measured after administration. The animals were treated with the different betaxanthin derivatives, and their gonads were measured from the loop region to the proximal region. The proximal gonad area is more swelled, as shown in Figure 2A, in comparison with that of the wild-type animal (N2), as it has been shown previously [24].

The eleven tryptophan-derived betaxanthins were assayed at 25 μM on a tumoral model in vivo following the method described in the bibliography [24]. Three of these treatments corresponded to the racemic mixtures since chiral pure precursors are not always available to check the effect of the mixture (Figure 2). All the treatments showed a reduction in the size of the tumoral gonad in a range between 31.4% and 43.0% (Figure 2M). The two most effective molecules reducing the tumor size were L-tryptophan-methyl ester-betaxanthin and L-tryptophan-benzyl ester-betaxanthin, which reduced the tumor size by 43.0% and 42.6%, respectively. This positive effect was followed by 5-bromo-DL-tryptophan-betaxanthin, tryptamine-betaxanthin, L-tryptophan-betaxanthin, serotonin-betaxanthin, DL-tryptophan-betaxanthin, D-tryptophan-betaxanthin, 5-fluoro-DL-tryptophan-betaxanthin, L-tryptophan-6-decarboxy-betaxanthin, and 5-hydroxy-L-tryptophan-betaxanthin which reduced tumor sizes by 39.9%, 38.1%, 37.0%, 35.8%, 35.7%, 34.9% 34.5%, 32.9%, and 31.4%, respectively (Table S1). After performing the screening of the novel compounds, the pigment L-tryptophan-methyl ester-betaxanthin has shown a 1.16 times higher effect with respect to that of the lead molecule L-tryptophan-betaxanthin.

### 2.4. Microarray Analysis

**Figure 3:**
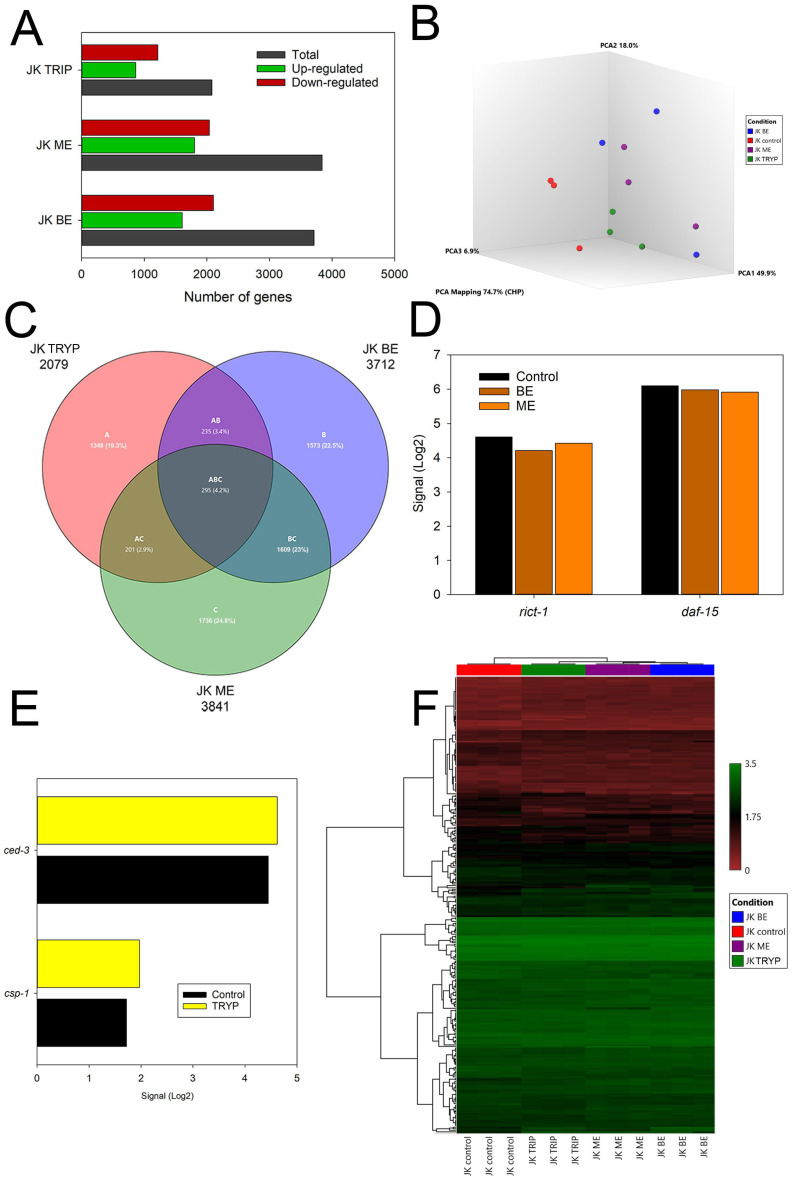
Differential gene expression analysis. (**A**) Genes up- and down-regulated by the treatments with the lead molecule tryptophan-betaxanthin, and the derivatives tryptophan benzyl ester-betaxanthin and tryptophan methyl ester-betaxanthin. (**B**) PCA analysis. (**C**) Venn diagram. (**D**) Expression levels of mTOR dysregulated genes in *C. elegans* treated with tryptophan benzyl ester-betaxanthin and tryptophan methyl ester-betaxanthin. (**E**) Expression levels of caspases dysregulated genes in *C. elegans* treated with tryptophan-betaxanthin. (**F**) Heatmap showing the triplicate assays for the conditions control, tryptophan benzyl ester-betaxanthin, tryptophan methyl ester-betaxanthin, and tryptophan-betaxanthin.

The differential expression analysis of mRNA was used to explore the effects of the betaxanthins obtained from tryptophan, tryptophan benzyl ester, and tryptophan methyl ester on *C. elegans* gene regulation. Tryptophan-betaxanthin supplementation to *C. elegans* produced alterations in 2079 genes; 1217 of them were down-regulated, while the rest were up-regulated (Figure 3A). The ester derivates had similar effects on mRNA expression, tryptophan-benzyl ester-betaxanthin altered 3712 genes, while tryptophan-methyl ester-betaxanthin modified the expression of 3841 genes. Principal component analysis (PCA) was used to visualize the distance association among the twelve samples according to the changes in expression. The PCA score plot indicates a clear separation between the control and the treatment samples (Figure 3B). Interestingly, the benzyl and methyl ester derivates are closer in the PCA plot, thus suggesting a common effect linked to the common structural feature. Moreover, as the Venn diagram (Figure 3C) reflects, the worms treated with these molecules showed more altered genes in common (1904) among them than with the tryptophan-betaxanthin. The ester derivate-treated animals showed a down-regulation of the key genes of the mTOR pathway, such as *rict-1* and *daf-15* (Figure 3D), when compared with those of the control animals. The clustering analysis of the heatmap also showed more similarities in gene expression between the esters (Figure 3F) than with tryptophan-betaxanthin and the control samples.

## 3. Discussion

Novel betaxanthins have been produced using L-tryptophan-betaxanthin as a lead molecule with a proven antitumoral capacity. These novel pigments have been biotechnologically produced and purified, and their properties have been characterized. When comparing 5-hydroxy-L-tryptophan-betaxanthin with L-tryptophan-betaxanthin, the former showed a shorter retention time, with around a five min shift, thus reflecting the lower affinity for the C-18 column matrix due to the higher hydrophilic nature of the novel molecule. However, all the other molecules showed longer retention times, corresponding to a more hydrophobic nature (Figure 1 and Table 1).

Color is one of the most important properties of natural betalains since they are plant pigments present in flowers and stems. The marked differences in color in closely similar structures, such as those containing fluorine or bromine, are noteworthy. They present differences in molar absorptivity coefficients, as also occurs with the derivatives in which the carboxylic group of the tryptophan moiety forms an ester. This shows the great influence that the nature of the functional groups has on the spectroscopic properties of the molecule (Figure S2 and Table 2).

These rationally designed betalains have been assayed in vivo in the tumoral animal model *C. elegans* (JK1466), showing a significant reduction in the size of the tumoral gonads for all of them (Figure 2 and Table S1). The structural modifications of the lead molecule, tryptophan-betaxanthin, either enhance or preserve the antitumoral effect reported previously for it [24]. The most effective molecules were L-tryptophan methyl ester-betaxanthin and L-tryptophan-benzyl ester-betaxanthin, the first betalains with ester functionalization, which were able to reduce the tumoral size by 43.0 and 42.6%, respectively. L-Tryptophan-betaxanthin, D-tryptophan-betaxanthin, and the racemic mixture DL-tryptophan-betaxanthin showed similar results, indicating that the configuration of the anomeric carbon did not influence the antitumoral activity and obtaining equivalent results to those of the lead molecule described in our previous study [24]. It is expected that the other two racemic mixtures, 5-bromo-DL-tryptophan-betaxanthin and 5-fluoro-DL-tryptophan-betaxanthin, would display similar results if their chiral isomers could be resolved and screened in an independent way.

The two most effective molecules described in this study present an ester group in their structure, being the only two structures assayed with this feature. These molecules present the carboxylic group of the tryptophan amino acid derivatized by the other substructures, such as a benzyl ester and a methyl ester group (Figure 1). Betalains esterification is an unexplored field in the bibliography, and these two betaxanthins are unique in their class.

The collected data seem to indicate that these modifications in the structure of tryptophan-betaxanthin could be relevant in the binding affinities between betaxanthin and the different targets involved in the cancer disease and in the interactions with the receptors or proteins, such as the Sirt1 protein, FTO protein, and PPARs [22,23,36].

The recent studies have used the same *C. elegans* tumoral model (JK1466 strain) to study the anticancer activity of some de novo designed metallodrugs [37,38]. These are chemical complexes analogous to cisplatin, where Platinum is substituted by novel metals. Cisplatin is one of the most used chemotherapeutic drugs for treating solid tumors in humans, although it has serious side effects, including kidney damage and hearing loss. The results in the same strain of *C. elegans* showed that cisplatin reduced the tumor size by 28.1 and 48.9% at 10 and 100 µM, respectively, indicating the response to drugs of clinical use and the suitability of the *C. elegans* model to test novel anti-tumoral candidate compounds [37]. Among the complexes with novel metals, the osmium(II)-arene complex called the 2a-complex showed a reduction of the tumor size by 32.3% [37] and a iridium(III) complex (OncoIr3) performed a decrease of 41.0% at its highest dose (100 μM) [38]. In comparison, the tryptophan-derived betaxanthins produced in this study reduced the tumor size by between 31.4 and 43.0%, showing similar effects to the organometallic drugs, including cisplatin.

About microarrays analyses, the mechanism of action (MOA) of tryptophan esters-betaxanthins might be, in essence, different to the MOA of the lead molecule tryptophan-betaxanthin, although their structures are similar. The ester derivates were able to down-regulate the genes of the mTOR pathway, such as *rict-1* and *daf-15* (Figure 3D, Figures S3 and S4), the *C. elegans* orthologs to the human RICTOR and RAPTOR, respectively. Indeed, the analysis of the differential gene expression of the JK1466 strain and N2 wild-type strain showed several genes up-regulated in the tumoral strain (Figure S5), namely *daf-15*, *sinh-1*, and *mlst-8* (C10H11.8). mTOR forms two multiprotein complexes mTORC1 and mTORC2 when it is associated with the RAPTOR or RICTOR proteins, respectively. Thus, the antitumoral effect of tryptophan benzyl ester-betaxanthin and tryptophan methyl ester-betaxanthin could be explained due the down-regulation of the m-TOR signaling pathway. As opposed to the ester derivates, tryptophan-betaxanthin supplementation caused limited alterations in the mTOR pathway. Nevertheless, its antitumoral mechanism of action seems to be related to apoptosis, as the genes encoding the caspases, *ced-3* and *csp-1*, are up-regulated (Figure 3D). CED-3 and CSP-1 are orthologs of the human caspases CASP3, CASP6, and CASP7 that promote apoptotic cell death. Therefore, the molecules able to rescue this loss of expression, such as tryptophan-betaxanthin, may be potential antitumoral drug candidates. Thus, microarray analysis showed that the underlying mechanism of the ester derivatives could be explained by their capacity to down-regulate the mTOR pathway.

## 4. Materials and Methods

### 4.1. Chemicals

L-tryptophan, D-tryptophan, DL-tryptophan, tryptamine, 5-hydroxy-L-tryptophan, 5-fluoro-DL-tryptophan, 5-bromo-DL-tryptophan, L-tryptophan-benzyl ester, L-tryptophan -methyl ester, serotonin, dopamine hydrochloride, 3,4-dihydroxy-L-phenylalanine (L-DOPA), sodium ascorbate, Luria-Bertani medium (LB), carbenicillin, chloramphenicol, kanamycin sulfate, and isopropyl β-D-1-thiogalactopyranoside (IPTG) were purchased from Merck KGaA (Darmstadt, Germany). Ammonium hydroxide, TRIzol, acetonitrile, and HPLC-grade water were obtained from Fisher Scientific (Leicester, UK). 

### 4.2. Production of Betalains by Cellular Factories

Tryptophan-derived betaxanthins were obtained by cellular factories. The enzyme 4,5-DOPA-extradiol-dioxygenase (DODA) from *Gluconacetobacter diazotrophicus* was expressed in *Escherichia coli*. The cells overexpressing the DODA enzyme was transferred to a reaction flask containing a solution of 7.6 mM L-DOPA or 7.6 mM dopamine hydrochloride to produce betalamic acid or decarboxy-betalamic acid [34], respectively. The bioreactors were supplemented with 10 mM sodium ascorbate to prevent oxidation. Finally, the corresponding amino acid or amine was added at 10 mM to yield tryptophan-derived betaxanthin. The bioreactors were maintained at 20 °C for 72 h.

### 4.3. Purification of Betalains

After 72 h, the bioreactors were centrifuged, and the supernatants were subjected to solid-phase extraction (SPE) in cartridges with C-18 matrices (Thermo Fisher Scientific, Waltham, MA, USA) to remove the substrates and by-products and to concentrate the obtained betaxanthin derivatives prior to individual purification procedures. The obtained molecules were eluted from the matrix with ethanol. Then, the ethanol was dried by rotary evaporation. The purification was accomplished by anion exchange chromatography using an Akta purifier (General Electric Healthcare, Milwaukee, Brookfield, WI, USA). The elutions were studied spectroscopically at 280 and 480 nm. Once the fractions were selected, SPE was performed again to remove the salt used in the purification step (NaCl). The purified pigment was frozen at −20 °C until use.

### 4.4. Absorbance Spectroscopy

To determine the molar extinction coefficients (ε) and the final concentrations of the tryptophan-derived betaxanthins, a Jasco V-650 (Easton, MD, USA) was used. The ε values were determined for all the species with an endpoint method that measures the degradation of the betaxanthin to betalamic acid or to 6-decarboxy-betalamic acid and the corresponding amino acid or amine by spectrophotometry. As a starting point, the spectrum of the novel betaxanthin in water was carried out, and then it was subjected to basic hydrolysis by adding an aqueous solution of ammonia 1:10. The degradation process was monitored for 30 min, performing cumulative spectra every 2 min. Since the molar absorptivity (ε) value of betalamic acid and 6-decarboxy-betalamic acid are known, 27,000 M^−1^·cm^−1^ and 29,000 M^−1^·cm^−1^ [34], respectively, calculations were made to obtain the ε values of the novel described betaxanthins using the values of the absorbance spectrum before and after the degradation of the pigment with ammonia. 

### 4.5. HPLC Analysis

A Shimadzu LC-10A (Kyoto, Japan) equipped with a PDA SPD-M10A detector was used to perform analytical HPLC separations. The wavelengths between 250 and 700 nm were recorded, obtaining the absorbance spectra of all the samples analyzed. Reversed-phase chromatography was carried out on a 250 × 4.6 mm Kinetex C-18 column (Phenomenex, Torrance, CA, USA) with a 5 µm particle size. The elution of the compounds was carried out by applying a linear gradient between two solvents; solvent A (initial conditions) was acidified water with 0.05% *v*/*v* TFA, and solvent B was composed of acetonitrile with TFA at 0.05%. The gradient was developed for 35 min from 0% *v*/*v* B to 35% *v*/*v* B using a flow rate of 1 mL·min^−1^, with an injection volume of 50 μL.

### 4.6. Analysis by Mass Spectrometry with Electrospray Ionization

The compounds were analyzed by mass spectroscopy in an Agilent 6220 TOF/Q-TOF MS spectrometer equipped with a dual ESI-APCI interface for obtaining exact mass determinations. The samples were positively ionized using a 3.5 kV capillary voltage. The drying gas was nitrogen at 350 °C, the flow was set at 11 L·min^−1^, and the nebulizer pressure was 40 psi. All the data were processed through MassHunter software vB.03.01 (Agilent Technologies).

The experimental mass (*m*/*z*) obtained after mass spectrometry analysis for each betaxanthin and the theoretical mass (*m*/*z*) of the tentative molecule were used for the calculation of Δppm by using Equation (1). The determination of the Δppm values served as a method of confirmation of the elemental composition, accepting values lower than 10 ppm for the unambiguous verification of the different compounds [39].

(1) $$\Delta ppm=\left(\frac{\mathrm{Experimental}\;\mathrm{mass}\;-\;\mathrm{Theoretical}\;\mathrm{mass}}{\mathrm{Experimental}\;\mathrm{mass}}\right)\times 1,000,000$$

Equation (1): calculation of Δppm for the verification of the tryptophan-betaxanthin-derived pigments.

### 4.7. C. elegans Culture Conditions and Strains

*C. elegans* strain JK1466 (*gld-1* (q485)/dpy-5 (e61) unc-13(e51)) was obtained from *Caenorhabditis* Genetic Center (CGC, St Paul, MN, USA), which is supported by the National Institutes of Health, Office of Research Infrastructure Programs (P40 OD010440). The nematodes were maintained at 20 ºC in solid nematode growth medium (NGM) [40]. The JK1466 strain was fed *E. coli* HT115 *gld-1* [41] to ensure all the worms had the *gld-1* gen knocked-down. The HT115 strain, which contains the homologous DNA sequence for the *gld-1*, was obtained from Source BioScience (sourcebioscience.com, accessed on 9 September 2021) from the library “RNAi Library (Ahringer)”. The experiments were performed in liquid S medium with age-synchronized animals using the bleach method [40].

### 4.8. Antitumoral Effect of Tryptophan-Derived Betaxanthins In Vivo

After synchronization, L1 larvae of the *C. elegans* strain JK1466 were collected and transferred to sterile 25 mL flasks containing 250 μL of a concentrated culture of *E. coli* HT115 *gld-1* 10x in M9 buffer, medium S supplemented with carbenicillin (30 μg·mL^−1^) and IPTG (1 mM), and 25 μM of each individually pure tryptophan-betaxanthin-derived pigment in a final volume of 5 mL. In the control experiments, the same volume of water was added instead of the betaxanthins. The flasks were kept under orbital shaking conditions at 20 °C for six days, and then the nematodes were analyzed to assure its viability, since the lifespan of this strain is of mean 7.92 ± 0.12 days. Dead or damaged animals were excluded from the assay [24]. Independent experiments were carried out for each of the tested compounds. The experiments were performed in duplicate; n > 50. 

### 4.9. Tumor Size Evaluation

A previously published tumor size measurement method was used to evaluate the potential antitumor activity of the novel analogous purified. For this, images of the animals were taken using the 20x lens on a Leica DM 2500 LED microscope fitted with a Leica DFC550 camera (Leica Microsystems, Wetzlar, Germany). Bright-field images were used to delimit and measure the area of each gonad using ImageJ software v1.53c [42]. Gonad sizes were measured from the loop region to the proximal region, including the area of the uterus when it was filled with tumor cells [24]. 

### 4.10. RNA Extraction and Microarray Analysis

After two days with the treatments in liquid medium, the worms were collected and cleaned with M9 buffer 10 times to ensure the elimination of *E. coli.* RNAi extraction was performed with TRIzol^®^ following the protocol of PureLink^TM^ RNA Mini Kit from Invitrogen (Carlsbad, CA, USA).

GeneChip WT Pico Reagent kit from Affymetrix was used to synthetized Ss-cDNA from 3.5 ng of each sample. The following steps of hybridization to GeneChip^®^ *C. elegans* Gene 1.1 ST Array Strip (Affymetrix, Santa Clara, CA, USA) with 26 unique sequences of each transcript were performed under the instruction of the manufacturer’s protocol. After scanning, the microarray data were processed using Affymetrix Transcriptome Analysis Console. Three independent RNA samples were employed. The control JK1466 strain and that treated with L-tryptophan-benzyl ester-betaxanthin or L-tryptophan methyl ester-betaxanthin were analyzed. The N2 strain was also analyzed and compared with the JK1466 strain.

### 4.11. Statistical Analysis

The statistical analysis of the data was performed using SigmaPlot 14.0 software (Systat Software Inc., San Jose, CA, USA). A one-way ANOVA test was employed with a significance level of *p* < 0.001.

## 5. Conclusions

Novel betaxanthins have been produced using the natural molecule L-tryptophan-betaxanthin as the lead molecule with a proven antitumoral capacity. These novel pigments have been biotechnologically produced and purified, and their properties have been characterized. These betalains have been assayed in vivo in the tumoral animal model *C. elegans* (JK1466), showing a significant reduction in the size of the tumoral gonads for all of them. The most effective molecules were L-tryptophan methyl ester-betaxanthin and L-tryptophan-benzyl ester-betaxanthin, the first betalains with ester functionalization described, which were able to reduce the tumoral size by 43.0 and 42.6%. Microarray analysis showed the capacity of tryptophan esters-betaxanthins to down-regulate the mTOR pathway. There was a difference in the effects shown by tryptophan-betaxanthin, whose MOA is related to caspase activation. The obtained results strengthen the role played by betalains in cancer chemoprevention by studying the mechanism of action underlaying the antitumoral effects of novel derivatives. This work highlights the wide possibilities of the chemical modification of phytochemicals by rational design to obtain derivatives with enhanced characteristics for possible application in cancer disease treatments.

## Figures and Tables

**Table 1:** Data analysis of tryptophan-derived pigments obtained using a biotechnological strategy. Retention times for HPLC analysis, chemical formula, and TOF exact mass determinations are shown.

Pigment	HPLC Analysis Rt (min)	Chemical Formula	TOF Exact Mass		
			Experimental *(m*/*z)*	Theoretical *(m*/*z)*	Δppm
L-tryptophan-betaxanthin	20.47	C_20_H_19_N_3_O_6_	398.1322	398.1347	6.2
D-tryptophan-betaxanthin	20.17	C_20_H_19_N_3_O_6_	398.1323	398.1347	6.0
L-tryptophan-6-decarboxy-betaxanthin	23.63	C_19_H_19_N_3_O_4_	354.1424	354.1448	6.7
Tryptamine-betaxanthin	22.95	C_19_H_19_N_3_O_4_	354.1464	354.1448	4.5
5-hydroxy-L-tryptophan-betaxanthin	15.31	C_20_H_19_N_3_O_7_	414.1277	414.1296	4.5
5-fluoro-DL-tryptophan-betaxanthin	21.53 21.78	C_20_H_18_FN_3_O_6_	416.1228 416.1231	416.1252 416.1252	5.7 5.0
5-bromo-DL-tryptophan-betaxanthin	24.63 24.88	C_20_H_18_BrN_3_O_6_	476.0426 476.0429	476.0452 476.0452	5.4 4.8
L-tryptophan-benzyl ester-betaxanthin	20.53	C_27_H_25_N_3_O_6_	488.1790	488.1816	5.3
L-tryptophan-methyl ester-betaxanthin	24.16	C_21_H_21_N_3_O_6_	412.1500	412.1503	0.7
Serotonin-betaxanthin	16.97	C_19_H_19_N_3_O_6_	370.1411	370.1397	3.7

**Table 2:** Absorbance data obtained for tryptophan-derived betaxanthins. Maximum wavelengths (λ_max_), molar absorption coefficients (ε), and absorbance spectral widths are indicated.

Pigment	Absorbance		
	λ_max_ (nm)	ε (M^−1^ cm^−1^)	Width (nm)
L-tryptophan-betaxanthin	477	42,000	64
D-tryptophan-betaxanthin	477	42,000	65
L-tryptophan-6-decarboxy-betaxanthin	474	49,000	61
Tryptamine-betaxanthin	473	36,000	59
5-hydroxy-L-tryptophan-betaxanthin	477	38,000	70
5-fluoro-DL-tryptophan-betaxanthin	479	43,000	61
5-bromo-DL-tryptophan-betaxanthin	479	55,000	62
L-tryptophan-benzyl ester-betaxanthin	475	45,000	68
L-tryptophan-methyl ester-betaxanthin	479	36,000	67
Serotonin-betaxanthin	474	43,000	68

## Data Availability

The microarray data from this publication have been deposited at the GEO database (NCBI) (https://www.ncbi.nlm.nih.gov/geo/, accessed on 16 June 2023) and assigned the identifier GSE235107.

## Supplementary Materials

The following supporting information can be downloaded at: https://www.mdpi.com/article/10.3390/ijms25010063/s1.
