# Supplementary material for: Design, Synthesis and Gene Modulation Insights into Pigments Derived from Tryptophan-Betaxanthin, Which Act against Tumor Development in Caenorhabditis elegans
Source: Int J Mol Sci. 2023 Dec 20;25(1):63. doi: 10.3390/ijms25010063 (PMC10778952; doi:10.3390/ijms25010063)
Supplement: Supplementary file 1 [file ijms-25-00063-s001.zip › ijms-2754837-supplementary.pdf]

Supplementary information

Design, Synthesis and Gene Modulation Insights into Pigments Derived from Tryptophan-Betaxanthin, which Acts against Tumor Development in *Caenorhabditis elegans*

Paula Henarejos-Escudero, Fernando F. Méndez-García, Samanta Hernández-García, Pedro Martínez-Rodríguez, and Fernando Gandía-Herrero

Department of Biochemistry and Molecular Biology A, Faculty of Biology, Regional Campus of International Excellence, Campus Mare Nostrum, University of Murcia, Murcia, Spain.

Table S1. *In vivo* measurements of tumor size. Statistical analysis: Kruskal-Wallis One Way Analysis of Variance on Ranks

	**[ ] (μM)**	**n**	**Tumor Area (μm^2)**	**S.D.**	**Reduction (%)**	***p* value *vs* control**
Control	25	243	14395.52	3434.07	0.0	
L-tryptophan-betaxanthin	25	89	9056.34	2215.15	-37.0	<0.001
D-tryptophan-betaxanthin	25	78	9372.10	1891.74	-34.9	<0.001
DL-tryptophan-betaxanthin	25	56	9247.86	3042.07	-35.7	<0.001
L-tryptophan-6-decarboxy-betaxanthin	25	89	9666.17	2088.68	-32.9	<0.001
Tryptamine-betaxanthin	25	99	8909.07	1944.82	-38.1	<0.001
5-hydroxy-L-tryptophan-betaxanthin	25	90	9871.99	2605.08	-31.4	<0.001
5-fluoro-DL-tryptophan-betaxanthin	25	80	9421.97	2309.92	-34.5	<0.001
5-bromo-DL-tryptophan-betaxanthin	25	81	8657.34	2142.69	-39.9	<0.001
L-tryptophan-benzyl ester-betaxanthin	25	87	8256.18	1901.74	-42.6	<0.001
L-tryptophan-methyl ester-betaxanthin	25	75	8212.28	1906.22	-43.0	<0.001
Serotonin-betaxanthin	25	107	9244.04	1564.24	-35.8	<0.001

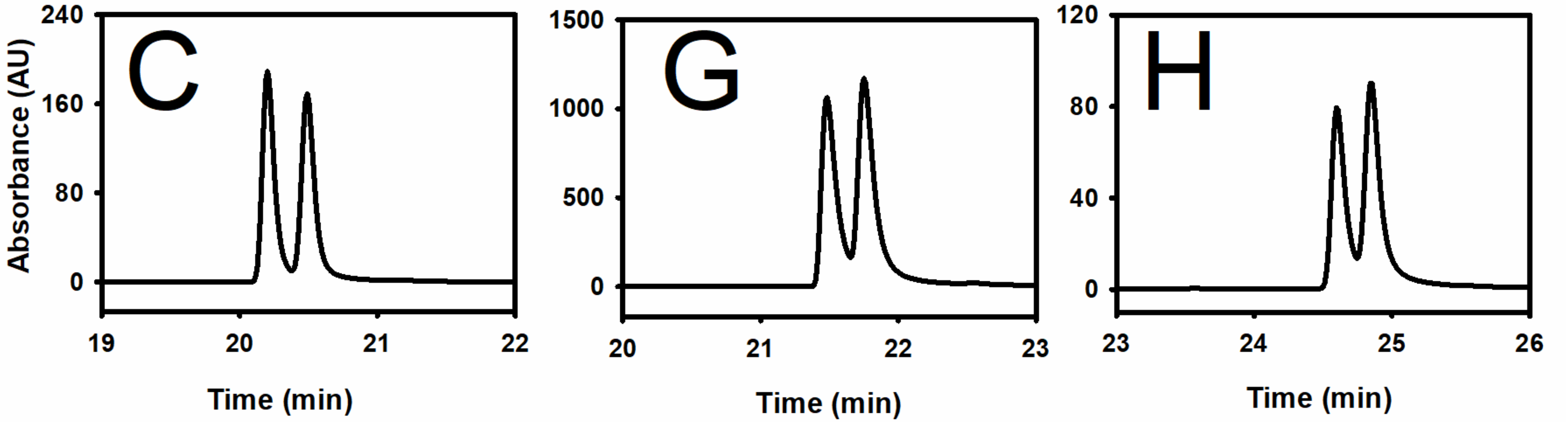

Fig. S1. Figure S1**.** Zoom of the peaks in the HPLC recording of Figure 1 where two peaks corresponding to the diastereoisomeric forms of the pigments derived from the racemic amino acids are shown in panels C, G and H. (C) DL-tryptophan-betaxanthin, (G) 5-fluoro-DL-tryptophan-betaxanthin, (H) 5-bromo-DL-tryptophan-betaxanthin. The injection volume was 50 μL.

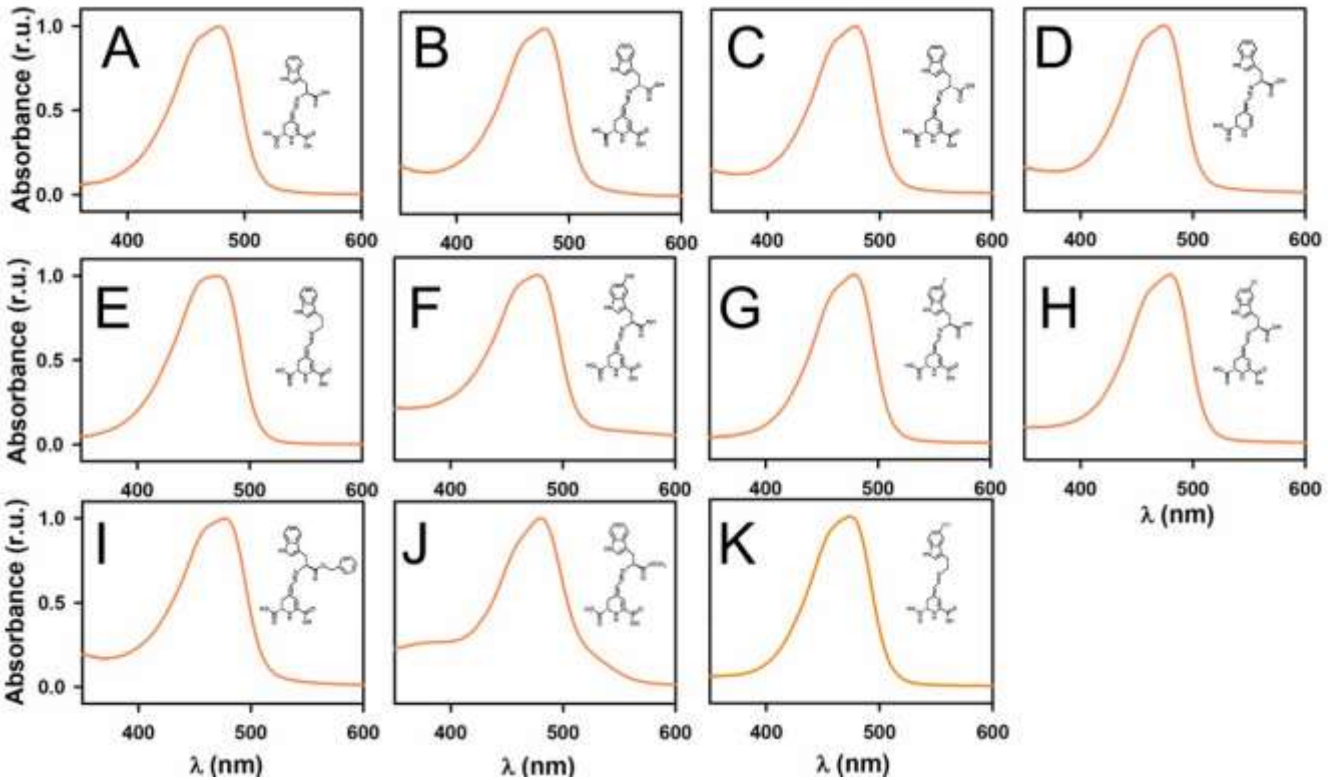

Fig. S2. Absorbance spectra for all the tryptophan-derived betaxanthins considered in this study. (A) L-tryptophan-betaxanthin, (B) D-tryptophan-betaxanthin, (C) DL-tryptophan-betaxanthin, (D) L-tryptophan-6-decarboxy-betaxanthin, (E) tryptamine-betaxanthin, (F) 5-hydroxy-L-tryptophan-betaxanthin, (G) 5-fluoro-DL-tryptophan-betaxanthin, (H) 5-bromo-DL-tryptophan-betaxanthin, (I) L-tryptophan-benzyl ester-betaxanthin, (J) L-tryptophan methyl ester-betaxanthin, and (K) serotonin-betaxanthin. r.u. relative units. Pigment structures are shown inset.

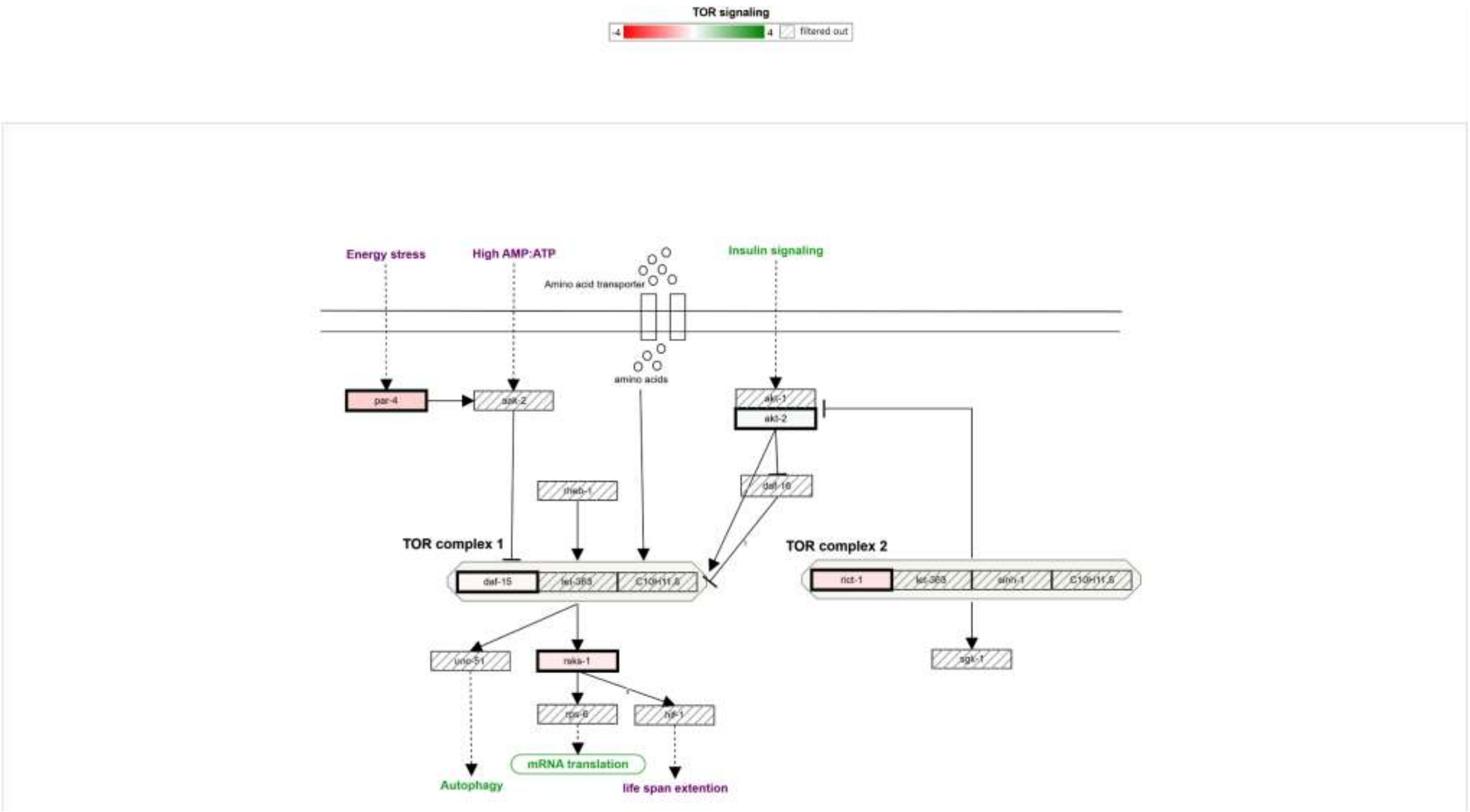

Fig. S3. Differential expression of mTOR pathway genes between tryptophan benzyl ester-betaxanthin and control treatments.

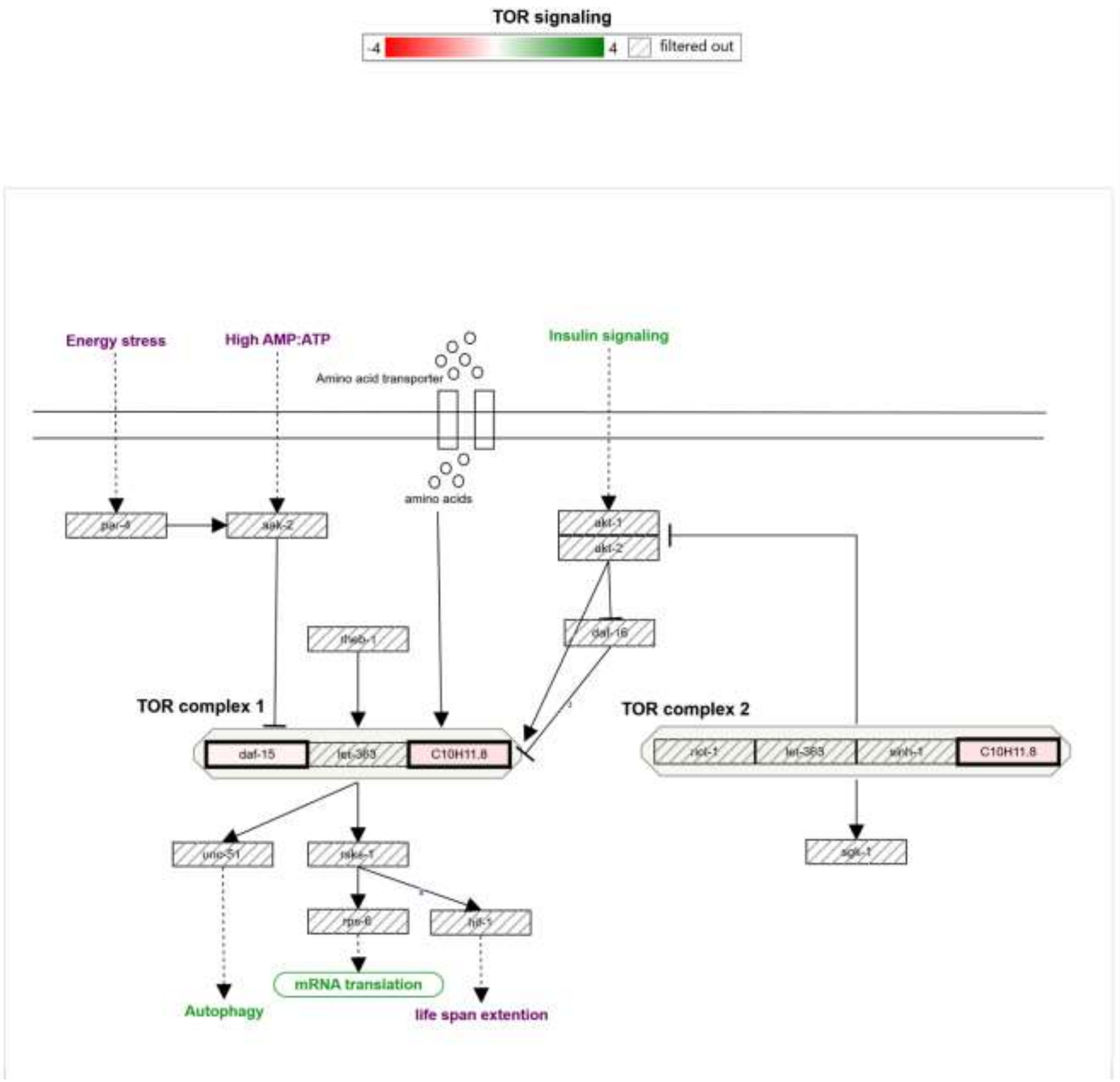

Fig. S4. Differential expression of mTOR pathway genes between tryptophan methyl ester-betaxanthin and control treatments.

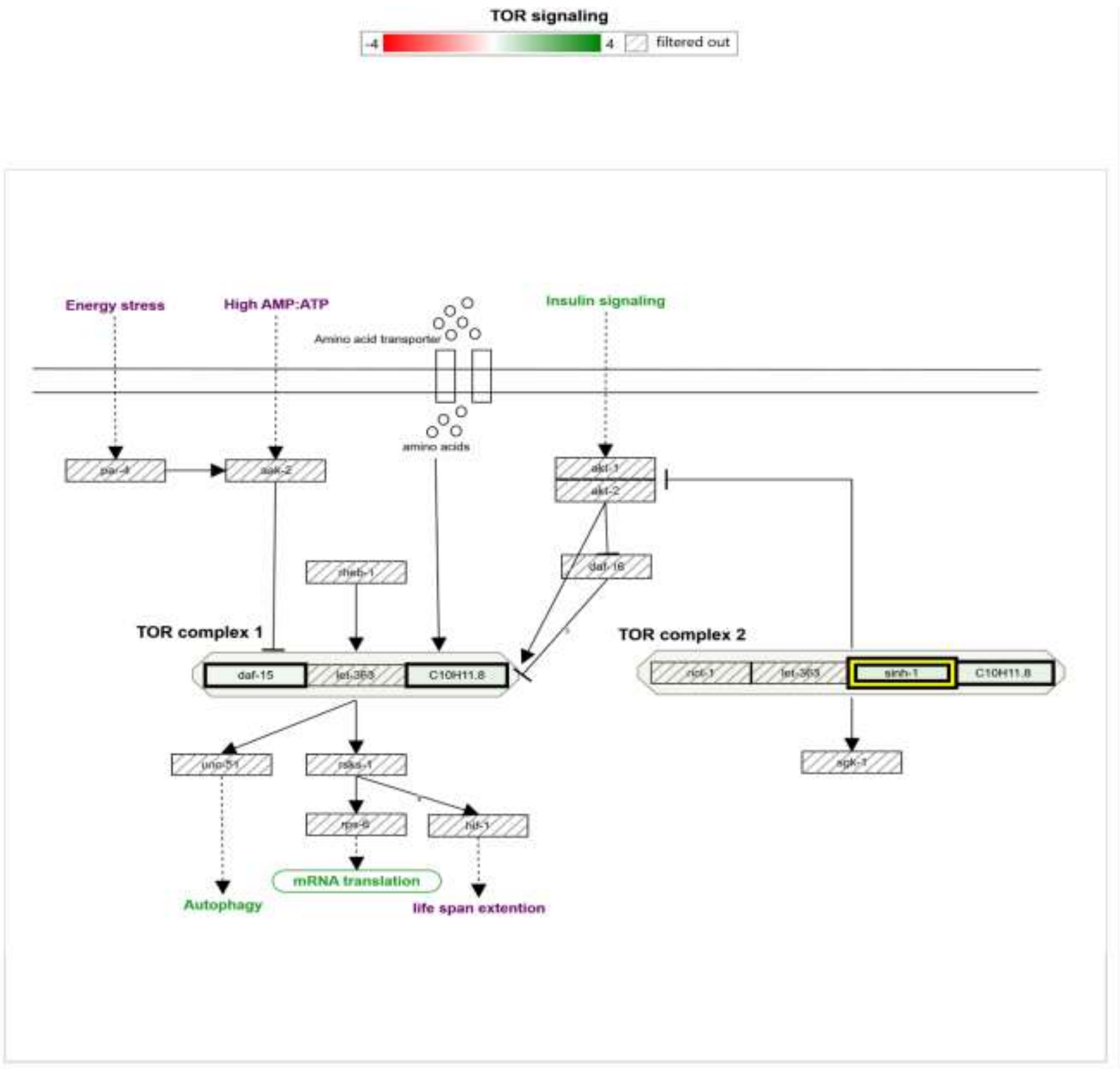

Fig. S5. Differential expression of mTOR pathway genes between JK1466 strain and N2 strain.
